# Schizophrenia in real life: courses, symptoms and functioning in an Italian population

**DOI:** 10.1186/1752-4458-6-22

**Published:** 2012-10-09

**Authors:** Maria Cristina Turola, Gloria Comellini, Anna Galuppi, Maria Giulia Nanni, Emanuela Carantoni, Chiara Scapoli

**Affiliations:** 1 Integrated Department of Mental Health and Drug Abuse, Local Health Agency, Ferrara, Italy; 2 Section of Child Neurology and Psychiatry, Children’s Hospital A. Meyer, University of Florence, Florence, Italy; 3 Section of Psychiatry, University of Ferrara, Ferrara, Italy; 4 Department of Biology and Evolution, University of Ferrara, Ferrara, Italy

**Keywords:** Schizophrenia, Course, Outcome, Hospitalization index, Remission, Personal and social functioning

## Abstract

**Background:**

In the general belief, schizophrenia is associated with the concepts of seriousness, incurability, dangerousness: this is incorrect. In recent decades, the interest in course studies increased and different trends emerged, not necessarily chronic, with the possibility of remission.

The plan of this research was to draw a picture of the schizophrenia syndrome in a specific geographic area, in the past and at present time: this allows to detect needs, weaknesses and strengths, for a better planning of future interventions.

**Methods:**

The course of all cases diagnosed as schizophrenia (N = 1,759) in the period 1978–2008, was retrospectively studied in the entire population of an Italian province by observing, for a mean period of 12 years per person, age at first psychiatric consultation, number and length of admissions for both acute symptoms and residential-rehabilitation programs, number of interventions in outpatients. The cases under treatment (N = 842), were evaluated in terms of symptoms, using the Brief Psychiatric Rating Scale, and in terms of functioning, using the Personal and Social Functioning Scale.

**Results:**

The disease course differs significantly between genders: males have an earlier age at first consultation (about 7 years earlier), higher admission rates, greater number of outpatient interventions and personal and social functioning significantly worse.

Hospitalization resulted often unnecessary: 23.1% of cases were never hospitalized and 67.2% spent less than one week per year in hospital.

A quarter of the cases meets the international criteria for remission and more than 75% are asymptomatic or mildly symptomatic; only 5.3% of cases shows severe symptoms. However, Personal and Social Functioning highlights, in about 1/3 of cases, relevant or serious problems mainly in Work and Relationships areas, whilst Aggressiveness is a serious problem only in 9%.

**Conclusions:**

In this population, schizophrenia in real life shows great individual variability in course, symptoms and functioning: in most cases nowadays it appears a less severe and chronic disease than in the past, but further improvements are needed on disability prevention and social inclusion.

## Background

This research originates from the daily work of mental health professionals and users, from their need to understand, to inform, to motivate themselves and improve.

In the general belief, schizophrenia is associated with the concepts of seriousness, incurability, dangerousness; the media often contribute to the stigma, and the same health care professionals do consider this type of psychiatric illness as alien and incomprehensible.

However, the mental health professionals, well knowing their patients, are aware of the changes which have occurred in recent decades, even though, between institutional requirements and needs of users, they often overshadow the favorable results, as they continuously deal with the most symptomatic or most difficult situations.

The schizophrenia syndrome is defined by a set of symptoms, following the indications of The International Classification of Diseases – ICD10 [1] and the Diagnostic and Statistical Manual of Mental Disorders – DSM IV-TR [2]; in the past, the diagnosis of schizophrenia has been often applied to a wide range of pathologies of different etiology (neoplastic, infectious, inflammatory diseases), in the absence of diagnostic tools to identify and differentiate. For example, in the second half of the nineteenth century, the Psychiatric Hospitals of some Italian regions (Emilia Romagna and Veneto) were crowded with people with evident psychotic symptoms - delusions, hallucinations, agitation, insomnia - due to deficiency of vitamin PP for indigence, hunger or exclusive feeding of corn; only the presence of skin lesions and the spread of an endemic disease allowed to diagnose this pathology as pellagra [3-5].

In other historical periods, particularly in the 70–80 years of last century, the diagnosis of schizophrenia was often avoided, as a label involving stigma, and was thus replaced with alternative diagnostic categories, such as paranoid personality disorder, or generic definitions of less compromising psychomotor agitation or anxiety; the diagnosis of schizophrenia, at the time, was raised only after several hospitalizations or in very severe cases. The first issue, in a retrospective research on schizophrenia, was therefore the precise definition of the sample of cases, including all cases with the disease and excluding the false diagnosis.

The schizophrenic syndrome has been the subject of a huge number of observations, studies and publications. For a long time, attention was centered on the description of symptoms, then on clinical cases and differential diagnosis, and then the therapeutic interventions prevailed. Courses and outcomes in the past were the subject of a limited interest because of the general belief that the evolution was always chronic and the outcome always incapacitating.

Until a few decades ago, the treatment of schizophrenia was mainly separation from society and seclusion in psychiatric hospital, where patients often spent their entire life, excluded from society and deprived of civil rights. The first half of the twentieth century in Italy, before and after two devastating wars, has been characterized by attempts of deemed “scientific” treatments of psychosis and of schizophrenia in particular, with the application, within Psychiatric Hospitals, of an heterogeneous typology of “therapies”: hot or cold baths, insulin coma, hyperthermia caused by pathogens such as malaria or typhoid, abuse of electroshock, surgical treatments such as lobotomy. The primary objective was to obtain ‘quiet’ patients, this was the only qualification that could allow the release from the hospital, if the relatives accepted the responsibility of a family housing. Healing was considered an unattainable goal, and the course of schizophrenia was heavily influenced and altered by bias due to institutionalization.

Only since the 70s of the last century, socio-cultural and therapeutic progress and specific socio-political choices led in some countries, including Italy, to deinstitutionalization, giving priority to the treatment in an outpatient setting; nowadays, this is the care model indicated by WHO [6]. This organization allows the observation of the schizophrenia course in the real life, without the bias of institutionalization.

In the following decades, the interest in course studies increased [7-10] and different trends emerged, not necessarily chronic, but dynamic, evolving and subject to change [11-13], with the possibility of remission [14].

The Schizophrenia Working Group on Remission [15] defines specific criteria for remission: improvement or disappearance of clinical symptoms, with any residual symptoms of low intensity not significantly interfering with personal well-being; clinical remission and absence of hospital admissions for at least 6 months; scores < = 2 in each item in the Scale for the Assessment of Positive Symptoms - SAPS or Scale for the Assessment of Negative Symptoms - SANS, or scores < = 3 in each item in Positive and Negative Syndrome Scale – PNSS or Brief Psychiatric Rating Scale – BPRS.

The study of outcomes, with the search of suitable measuring instruments [16], increasingly takes into account not only the level of symptoms but also the personal and social functioning, and the personal well-being. In recent years, the aim of treatment focused not only on remission, but also on recovery, with the return to premorbid conditions, development of new skills and attitudes, feeling of living a satisfying and contributing life [17,18]; the attention to the users opinion is growing[19,20].

The plan of this research was to draw a picture of a schizophrenia-diagnosed population, in the past and at present time, in a specific geographic area, the province of Ferrara - Italy, where the outpatient model of care has been applied for over 30 years. The aim was to know the extent of the disease, to measure, indirectly, the work of services in previous years and to try to detect needs, weaknesses and strengths, for the better planning of future interventions.

## Methods

### Setting

The Province of Ferrara is in the Emilia-Romagna Region- Italy, and has a population of 351,463 inhabitants, 168,205 males and 183,258 females, stable, with an immigration rate around 5%; the adult population (≥ 18 years) consists of 309,265 inhabitants: 146,422 males and 162,843 females. The social and demographic structure of the population did not change significantly in the last thirty years, except for a decline in births and a raising of the mean age.

**Figure 1:**
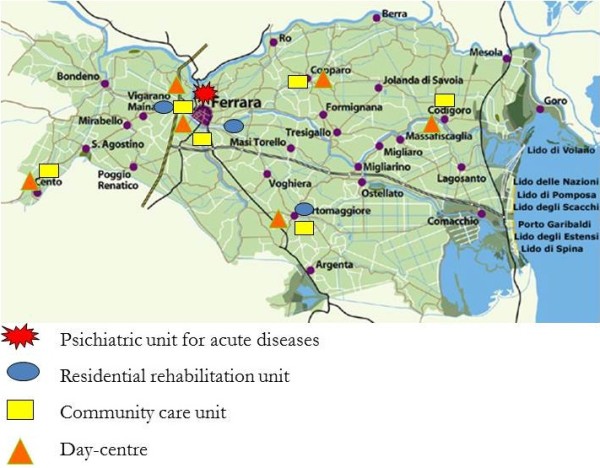
Province of Ferrara: structure of the Mental Health Department.

The entire adult population of the province is included in the caseload of the Ferrara Mental Health Department, which is organized through: six Community Mental Health Centers, six Day Centers, two hospitalization units for acute illness (one for only voluntary hospitalizations, the other for both voluntary and compulsory admissions- 30 beds in total), and three residences (60 beds in total) dedicated to individual rehabilitation programs lasting 3–6 months (Figure 1). The structure of the Department did not change significantly during the observed period, in spite of a gradual reduction of the number of the Community Mental Health Centers and of the resources of the staff, related to economic factors. There are no private psychiatric clinics in the province.

Users’ associations and relatives’ associations collaborate with the Department in socializing activities.

Since 1978, all areas have always interacted through an interconnected network: the main reference for patients is the Community Mental Health Center, with customized programs of care. The treatment programs may include: visits, interviews, drug therapy (oral, injection, and long-acting), psycho-education, psychotherapy, expressive therapies, family therapy, self-help groups and rehabilitation interventions of various kinds. Electroshock is always excluded.

The activities of the entire Department have been computerized and monitored since the 80s, with database systems (i.e. GESAP, SIPER, Ippocrate) registering the personal data of the cases and the data of all interventions, outpatient and inpatient, in line with other Italian case-registers [20]: that allow us to follow the path of individual patients over time.

### Participants

For this study we selected all the subjects - outpatient or inpatient - cared for in the Ferrara Mental Health Department (N =1,851), who received a diagnosis of schizophrenia in the period from 1979 to 2008.

We excluded the subjects who had lived in a psychiatric hospital continuously for more than three years and still lived in the psychiatric hospital in 1978, whilst we included the subjects discharged and cared for at home.

Data sources were: the department data bases, the medical records and all information received by patients, family members, caregivers and professionals. The Authors have personally collected all the data, in the period 2008 (recruitment, diagnostic checking) – 2010 (end of the evaluation of the cases in treatment).

### Design of the study

**Figure 2:**
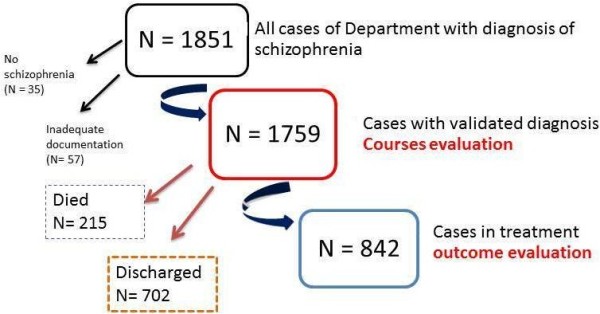
Flow chart of the research.

(Figure 2) Each case was checked for correspondence, current or in the past, with the ICD 10 or DSM IV- TR criteria for schizophrenia (F20 and 295 codes respectively), using the clinical records and the evaluation of the professional care provider. A total of 35 cases were excluded because they did not really meet the schizophrenia diagnosis criteria; other 57 subjects were excluded for disputable or inadequate documentation.

### Study of courses

A total of 1,759 cases were included in the first phase of the research: the retrospective study of individual paths and courses of all patients, conducted by recording the following parameters:

1. Age at the first consultation in Ferrara Psychiatry Department, assumed as Time 0 (T0);

2. Site of first consultation: community health service or hospital;

3. Gender;

4. Observation/follow-up time. In the discharged or dead cases, this time was calculated from T0 to the last consultation or reliable information;

5. Number and length of hospital admissions, respectively: (a) in Acute Units, (b) in Rehabilitative-Residential Units and (c) total;

6. Number of Community Mental Health Intervention per year, where “intervention” means all kind of consultation: with doctors, nurses, caseworkers, psychologists or rehabilitation therapists;

7. Day Center programs;

8. Number of cases discharged for having completed successfully the treatment program or having decided to stop it; all the other cases are considered still in treatment, even if living in protected or nursing home.

9. Number of deaths

For each subject, we calculated the following Indexes, which allow comparison among subjects with different observation time:

Total Hospitalization Index (THI) = average number of hospitalization days per observation year;

Acute Hospitalization Index (AHI) = average number of acute hospitalization days per year;

Rehabilitating/Residential Index (RRI) = average number of residential rehabilitation days per year.

### Study of symptoms and functioning

The second phase of the research was performed on patients treated in 2009 in the Department (N = 842), considering both symptoms and personal/social functioning.

The evaluation of symptoms was conducted using the Italian version of the Brief Psychiatric Rating Scale - BPRS in its 24-item 4.0 version [21-23]: each item is rated on a seven-point scale from 1 (absence of symptoms) to 7 (extremely severe symptoms); the total score is between 24 and 168. BPRS includes four subscales: Positive Symptoms (score range 5–35), Negative Symptoms (score range 7–49), Anxiety/Depression (score range 6–42) and Mania/Disorganization (score range 6–42). This ad hoc evaluation was made by the treating psychiatrist, taking into account the clinical status of the last three months; in case of significant changes in clinical status, we decided to consider the worst condition in the period.

The Functioning evaluation was performed using a modified version of Social and Occupational Functioning Assessment Scale –SOFAS of DSM IV-TR, named PSP - Personal and Social Performance Scale [24,25] or FPS - Personal and Social Functioning Scale[26,27].The scale measures the level of functioning in four areas: Work and Socially Useful Activities, Family and Social Relationships; Self Care and Health Care; Bothering and Aggressive Behaviors.

The information was gathered by the Authors, interviewing the case-managers, mainly nurses, and referring to the three months preceding the interview; in case of hetero/self-aggression or evident social disturbance, the assessment covers the period of one year. Serious and repeated self-injurious or hetero-aggressive events are taken into account in the evaluation, even if that occurred long before. The information collected about the four Areas was then converted into 5-level scales of difficulties(absent-mild-evident-relevant-serious, expressed as numeric values from 0 to 4 for processing). In the case of serious and repeated aggression, it is possible to assign the level 5 = extremely serious; furthermore, the functioning levels in the Bother/Aggressiveness Area influence the global evaluation more than the other three Areas. The ratings on the different areas, according a specific reading frame, form the global evaluation, expressed on a scale from 0 (the worst functioning) to 100 (the best possible functioning), subdivided in 10 equal intervals. The scoring of the PSP/FPS was performed, independently, by two psychiatrists blind to other information, such as the parameters of course and the symptom’s evaluation, and specifically trained, about this methodology, as evaluators. They discussed among each other the inconsistencies in the ratings and assigned an intermediate score, when the different evaluation persisted.

### Statistical analysis

The statistical analysis was carried out using STATA10®, Winstat® for Excel and STATISTICA 7.1®: in addition to the descriptive statistics, Kolmogorov-Smirnov goodness of fit tests was computed for each variable to assess whether the variables were normally, Gaussian distributed. Since most of the variables were not normally distributed, the non-parametric Mann–Whitney test was applied to compare groups. Data were described both with mean ± standard deviation (S.D.) and by median and inter-quartile range (I.R.).

The non-parametric Spearman test was used to express the relationship between variables. Given the present sample-size, even small or trivial effects were likely to produce statistically significant results; thus, we corrected for the effect-size by following Cohen [28] and we fixed a coefficient ≥ 0.80 as large; a coefficient ≤ 0.4 as small and the remaining as moderate.

Cluster Analysis was applied as an exploratory data analysis tool to find out simply structures within the data. The pair-wise distances matrix of the analyzed parameters was constructed using Euclidean distances and the distances between two clusters was calculated by using the Unweight Pair-Group Method with Arithmetic Means (UPGMA) [29].

## Results and discussion

In this study, the purpose was to get a description of the schizophrenia-diagnosed population in our area, of the course after the first consultation and of the symptomatic and functional conditions at present time.

### Course

The courses of the syndrome were studied in 1,759 subjects, 927 males and 832 females.

Males are more than females, with a gender ratio M/F = 1.11; in the general population of the Ferrara province the gender ratio in the range of age 0–99 is 0.92, but it is 1.29 in the range of age 18–60, where the peak of expression of the syndrome lies. The M/F ratio of the schizophrenia affected sample is thus in line with the ratio of general population.

#### Time of observation

The mean observation time is 12.9 years, with a standard deviation of 7.5. This long time of observation allows to adequately detect the phases of a syndrome characterized by a very high individual variability, with remissions and relapses.

#### First consultation

The first contact with the Mental Health Department, assumed as T0, occurred in outpatients (planned or urgent) for 985 cases (55.9%) and in hospital (always in emergency) for the remaining 774 cases (44.1%). The first consultations in emergency often led to hospitalization: better information and a better organization of CMHC could decrease the percentage of first contact in emergency, avoiding perhaps some of the subsequent hospitalizations.

#### *Age at* T0

The mean age at T0 is 37.5 ± 14.42. At T0, males are younger than females (34.0 ± 13.14 versus 41.5 ± 14.73): a significant difference, with p < 0.0001. This high mean age, with range 31.38-42.2 did not change over the time. The first consultation in the public Mental Health Department - T0 - does not correspond to the onset of the disease, which is located at an earlier time; we can consider T0 more as an acknowledgment of the severity of the disease, which can no longer be ignored or underestimated, or treated only by a single private specialist. The WHO review of 1997 [30] evidenced, in 7 out of 9 studies, a later onset of schizophrenia in females, and a high mean age in females at first contact with the psychiatric institutions in all the 11 studies considered.

**Figure 3:**
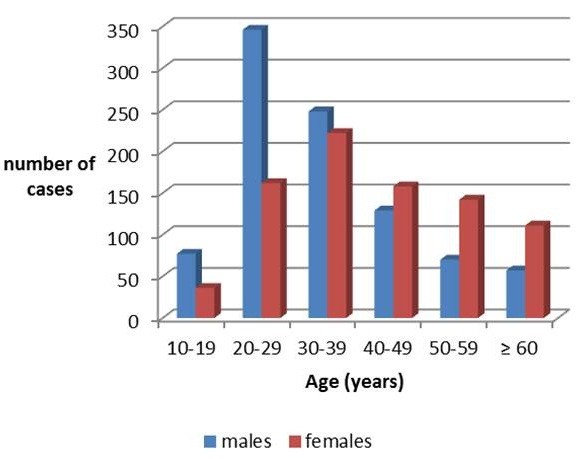
Age at T0 in the total sample of patients.

**Figure 4:**
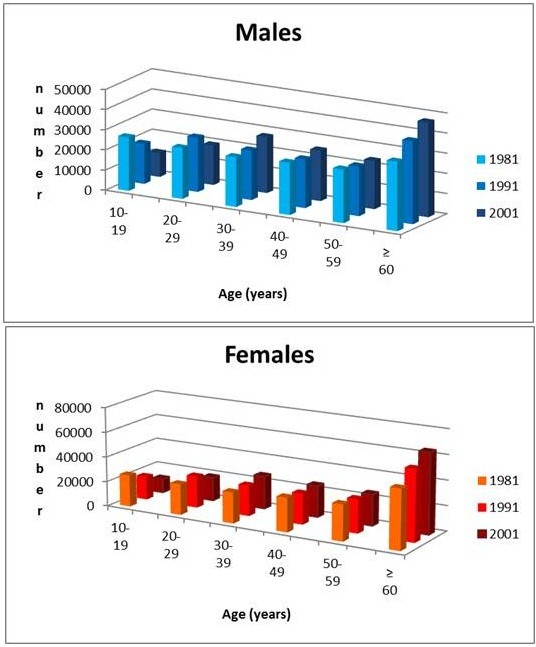
Frequency distribution of age in the general population.

Figure 3 shows the frequency distribution, gender-disaggregated, of age at T0 in the patient’s sample; Figure 4 shows the frequency distribution of age in the general population of the Province in the census of 1981, 1991 and 2001.

#### CMHC interventions

**Figure 5:**
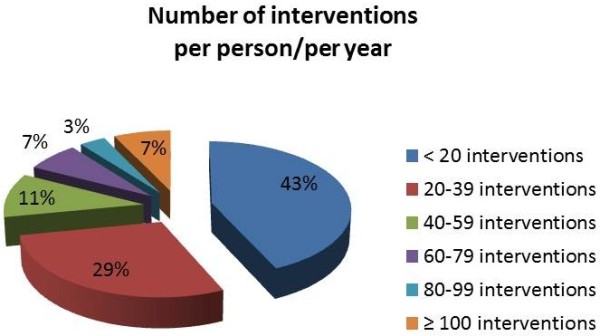
Number of CMHC intervention per person per year.

After the first consultation, subjects undertake a personalized program of treatment, which is done mainly in the CMHC. The average number of CMHC interventions per person per year is 38.0 ± 50.59, significantly higher (p = 0.009) in males (42.5 ± 58.47) than in females (32.0 ± 36.46). This data is referred to the last three years: in the past, the number of interventions per person was higher, and we find a descendent trend, not completely in line with a recommended management of schizophrenia and corresponding to the decline in economic resources invested in the CMHC. Taking into account any type of intervention, 43.2% of the subjects require less than 20 interventions per year, whereas only 7.2% of cases require more than 100 interventions per year (Figure 5). Therefore, in most cases the diagnosis of schizophrenia does not lead to a very high workload per single case; the workload is rather due to the amount of cases assisted for a long period of time and to their stratification.

#### Day center programs

388 people (19.9%) followed in their life-time a day center program: these data are largely underestimated, because the computerized registration of the day center activity started in the last ten years, and some of the previous case registers were lost; the anamnestic reconstruction is impossible in the older cases. The activity of the day centers is further underestimated because many interventions carried out into the general society, such as sports or theater, are often not registered as clinical interventions.

#### Hospitalization

The hospitalization is proposed in case of acute crisis (Psychiatric Ward in General Hospital), when the home care is impossible or ineffective, or in case of rehabilitating residential programs (Rehabilitation Unit).

The total number of admissions, in the whole sample, is 8,457; the percentage of non-voluntary admissions is 14.9% (N =1,257).

Table 1 shows the descriptive statistics of all hospitalization’s parameters, in the entire sample.

**Table 1:** Age at T0 and parameters of hospitalization in the entire sample (N = 1,759)

	**Total**		**Males**		**Females**		**P-value Mann–Whitney**
	**Mean (S.D.)**	**Median (IR)**	**Mean (S.D.)**	**Median (IR)**	**Mean (S.D.)**	**Median (IR)**	
Age at T0	37.5 (14.42)	34 (22)	34.0 (13.14)	31 (17)	41.5 (14.73)	39 (22)	< 0.0001
Years of observation	12.9 (7.56)	13 (10)	12.9 (7.72)	13 (12)	12.9 (7.39)	13 (10)	0.760
Total number of admissions	4.8 (11.26)	2 (4)	5.7 (13.26)	2 (5)	3.8 (8.40)	1 (4)	< 0.0001
Length of admissions (days)	213.3 (720.44)	31 (95)	246.4 (792.55)	39 (112)	176.3 (628.88)	25 (87)	< 0.0001
Admissions for acute crisis (number)	3.8 (8.37)	2 (3)	4.4 (9.54)	2 (3)	3.0 (6.76)	1 (3)	< 0.0001
Admissions for acute crisis (length/days)	63.9 (145.01)	24 (66)	76.7 (176.79)	28 (69)	49.7 (96.29)	21 (55.5)	< 0.0001
Rehabilitation -residential programs (number)	1.0 (3.96)	0 (1)	1.2 (4.87)	0 (1)	0.8 (2.57)	0 (0)	0.030
Rehabilitation-residential programs (length/days)	149.6 (660.54)	0 (2)	170.3 (715.53)	0 (20)	126.5 (592.88)	0 (0)	0.005
Total Hospitalization Index - THI	15.7 (39.84)	2.8 (10.2)	17.9 (41.91)	3.3 (13.8)	13.3 (37.29)	2.3 (8.35)	< 0.0001
Acute Hospitalization Index - AHI	6.1 (13.49)	2.2 (6.2)	7.2 (15.73)	2.6 (7.2)	4.9 (10.34)	1.9 (5.45)	< 0.0001
Rehabilitation-residential Index - RRI	9.8 (35.48)	0 (0.4)	10.8 (36.35)	0 (2)	8.6 (34.47)	0 (0)	0.029

**Figure 6:**
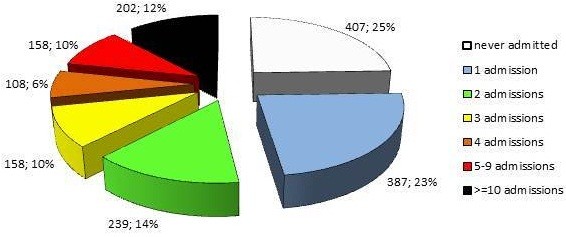
Number of admissions per person.

407 individuals (23.1%) were never hospitalized in a psychiatric ward in the entire course of their disease, whereas 1,352 had one or more admissions; 387 cases (22.0% of the total sample) had only 1 psychiatric admission, 495 cases (28.1%) had from 2 to 4 admissions, 158 cases (9.0%) had from 5 to 9 admissions and only 202 subjects (11.5%) had more than 10 psychiatric admissions in their lifetime (Figure 6). The number of admission in Psychiatric Ward for acute symptoms, in the single cases, may be considered as an indicator of the crisis episodes in the observation period, indicator largely underestimated because most of the crisis is treated in outpatient. It is difficult to compare this results with the “classical” studies on schizophrenia course [7,10], for the lack of specific data on hospitalization during the follow-up; re-hospitalization, in our sample, results lower than in past long-term studies [31].

The total duration of hospitalization, i.e. the number of days of hospitalization per subject, shows a great variability, with a very wide range, both for the great individual variability and in presence of different hospitalizations: acute symptoms crisis require different wards and times compared to a rehabilitation residential program.

We therefore examined separately the admissions for acute crisis and the admissions for rehabilitative residential programs. Patients with a diagnosis of schizophrenia had, on average, 3.8 admissions for acute crisis, and spent 63.9 days of their life in a ward for acute crisis; on average, they had only 1 admission for rehabilitative residential program, with a duration of 149.6 days and a greater impact on their individual life and on social costs.

But the “theoretical average patient” does not help us to focus on single cases: the Hospitalizations Indexes allow for the comparison between cases with different courses and trends and give more detailed information on individual cases.

How many days a schizophrenia attained patient spends in hospital per year, interrupting the normal course of his/her life?

**Figure 7:**
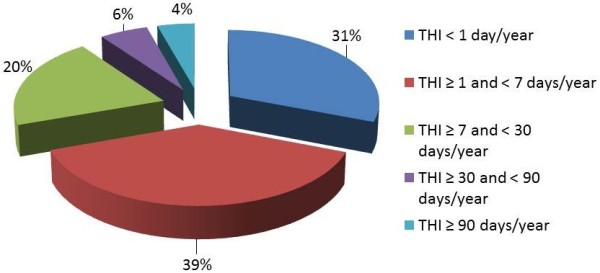
Total Hospitalization Index: percentage distribution.

A total of 542 cases (30.8%) spent in hospital, in the course of the disease, less than one day per year (THI < 1), 683 subjects (38.8%) less than a week per year (THI ≥ 1 and < 7); 357 subjects (20.3%) were hospitalized from a week to a month, 100 subjects (5.7%) from one to three months and 77 cases (4.5%) spent in hospital more than 90 days per year. We can affirm that in most cases the hospitalization did not significantly interrupt the normal course of life (Figure 7).

The Indexes have proved to be useful also on detecting individual trends, analyzing the individual cases during the treatment program. For example, an index of hospitalization decreasing over the years may indicate a clinical stabilization or a better compliance or an improving personal autonomy. An increasing Index may indicate an high level of symptoms or a poor compliance or an increasing disability of users, or serious difficulties in relationships within the family or within the social environment.

**Figure 8:**
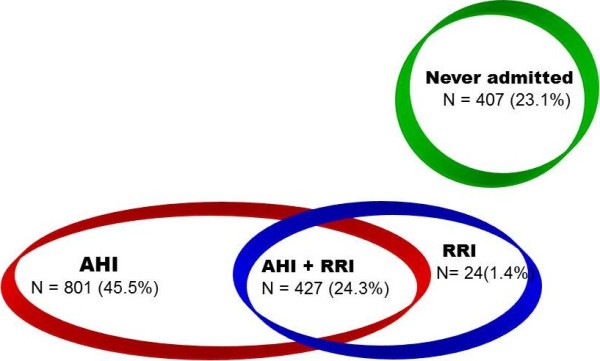
Needs for hospitalization.

AHI and RRI Indexes highlight different needs of hospitalization: only for acute crisis (high AHI), only for rehabilitation programs (high RRI) or both (high AHI and RRI) (Figure 8); the Indexes are thus indicators of the needs of this population in the short-medium period, allowing for a better planning of the Mental Health Services. They can be instrumental in preventing repeated hospitalizations, detecting the cases with high AHI related to low compliance, frequent interruptions of the treatment and relapses with strong acute symptoms. In these cases it is mandatory to spend more time and attention to inform, to search for consensus and cooperation and to improve the adherence to the treatment program.

A small heterogeneous group of subjects with high RRI requires instead a case by case analysis of needs, problems, skills, family and social environment, with the aim of limiting the duration of admission and implement rehabilitation programs outside rather than in hospital.

The analysis of all the parameters of admission indicates that the treatment of schizophrenic syndrome in most cases and most of the time is achievable outside the hospital.

### Discharges and deaths

Over the past thirty years, 702 people were discharged for having completed the treatment program or having chosen to stop it. A total of 215 cases, 92 females and 123 males, died: 14 cases died by suicide, 2 for accidents, 2 cases killed by relatives; for the remaining 197 cases we do not have detailed information, if not the generic definition of death by natural causes. The mean age at death was 51.89 ±15.7 in the males, 64.28 ± 14.02 in the females. The percentage of deaths in this sample is low, if compared to past long-term studies [10,32].

### Symptoms and Functioning

In 2009, 842 persons - 486 males and 356 females - followed a treatment program in the public Department of Mental Health.

Table 2 shows the descriptive statistics of age at T0 and all the hospitalization parameters, total and gender disaggregated, in the treated group.

**Table 2:** Age at T0 and parameters of hospitalization in the cases under treatment (N = 842)

	**Total**		**Males**		**Females**		**P-value Mann–Whitney**
	**Mean (S.D.)**	**Median (IR)**	**Mean (S.D.)**	**Median (IR)**	**Mean (S.D.)**	**Median (IR)**	
Age at T0	33.1(11.54)	31(16)	30.7 (10.48)	29 (13)	36.3(12.14)	35 (16)	< 0.0001
Years of observation	13.4 (7.36)	13 (10)	13.5 (7.57)	13 (11)	13.3 (7.08)	13 (9)	0.892
Total number of admissions	7.5 (14.86)	3 (6)	7.9 (16.88)	4 (7)	6.1 (11.28)	3 (5)	0.015
Length of admissions (days)	300.0 (731.57)	64 (178)	313.1 (744.65)	68 (216)	282.1 (713.98)	58 (148)	0.071
Admissions for acute crisis (number)	5.3 (10.79)	2 (4)	5.9 (11.78)	3 (5)	4.5 (9.22)	2 (3)	0.003
Admissions for acute crisis (length/days)	88.3 (182.18)	43 (80)	99.9 (213.57)	47 (83)	72.5 (126.03)	35 (68)	<0.004
Rehabilitation -residential programs (number)	1.8 (5.43)	0 (2)	2.0 (6.53)	0 (2)	1.5 (3.39)	0 (1.5)	0.499
Rehabilitation-residential programs (length/days)	212.4 (630.88)	0 (87)	214.2 (614.98)	0 (100)	209.8 (652.82)	0 (75)	0.465
Total Hospitalization Index THI	22.1 (46.94)	5.2 (16.7)	21.8 (41.57)	5.6 (19.4)	22.5 (53.46)	4.9(13.6)	0.159
Acute Hospitalization Index AHI	7.3 (13.0)	3.6 (7.0)	7.9 (13.72)	3.9 (7.8)	6.5 (11.92)	3.1 (6.3)	0.013
Rehabilitation-residential Index RRI	14.9 (39.72)	0 (7.4)	14.3 (34.92)	0 (9)	15.8 (45.52)	0 (6.2)	0.415

The cases under treatment significantly differ from the total sample (p < 0.0001) for a lower age at T0 (and significantly lower in males than in females) and higher Hospitalization Indexes THI, AHI, RRI. It is likely that individuals with early-onset or increased need for hospitalization, maintain contacts with the Department, while subjects who are treated only outside the hospital are more easily discharged.

#### Symptoms

How are our patients nowadays? What are their levels of symptoms?

The descriptive statistics of BPRS total score and of the BPRS subscales are represented in Table 3.

**Table 3:** Descriptive statistics of BPRS and PSP/FPS scores

**Scale**	**Total**				**Males**				**Females**				**P-value Mann–Whitney**
	**Mean**	**S.D.**	**Median**	**IR**	**Mean**	**S.D.**	**Median**	**IR**	**Mean**	**S.D.**	**Median**	**IR**	
**Total BPRS**	**58.3**	**20.98**	**56**	**30**	**59.7**	**21.66**	**58**	**34**	**56.4**	**19.89**	**54**	**27**	0.031
BPRS Anxiety-Depression	14.6	6.08	14	9	14.4	6.29	13	10	14.9	5.79	14	8	0.075
BPRS Positive Symptoms	13.9	6.41	13	10	14.4	6.65	14	10	13.1	6.01	13	9	0.011
BPRS Negative Symptoms	16.9	7.45	16	10	17.5	7.45	16	11	16.1	7.38	15	10	0.003
BPRS Mania/Disorganization	13.0	6.13	11	9	13.5	6.32	12	10	12.3	5.80	11	7	0.007
**Total PSP/FPS**	**55.0**	**22.47**	**54.5**	**32**	**52.1**	**22.53**	**50**	**33**	**58.9**	**21.82**	**58**	**28**	<0.0001
Work/Social utility	2.1	1.55	2	3.5	2.3	1.56	2.5	3	1.9	1.50	2	2.5	0.001
Personal relationships	1.9	1.33	2	2	2.1	1.29	2	2	1.7	1.35	2	3	<0.0001
Personal and Health care	1.6	1.33	1.5	3	1.7	1.35	2	3	1.4	1.26	1	2.5	<0.0001
Bother/Aggressiveness	0.6	1.11	0	1	0.7	1.19	0	1 .5	0.5	0.98	0	0.75	0.024

The mean total score is 58.3 ± 20.98, corresponding to a level of symptoms between very mild and mild. A total of 221 cases (21.24%) meet the International Criteria for Remission and over 78% of the cases, even if not satisfying the criteria, are asymptomatic or mildly symptomatic, using a broad range of treatment. The percentage of cases with severe symptoms is low (5.3%).

**Figure 9:**
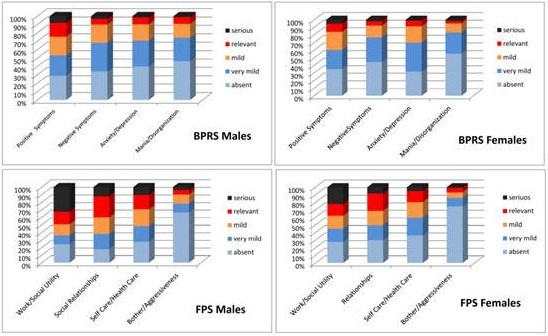
Frequency distribution of BPRS subscales and PSP/FPS subscale in males and females.

Figure 9 shows the frequency distribution of BPRS scores, total and subscales, in males and in females. Males are significantly more symptomatic than females both in the total score and in the subscales of Positive Symptoms, Negative Symptoms and Mania/Disorganization.

#### Functioning

What is the level of personal and social functioning? This assessment is always one-sided, strongly influenced by environmental and cultural factors; in this research, the functioning evaluation concerns the areas of work and social utility, the relationships, the personal and health care, the social disturbance and the aggressiveness.

The descriptive statistics of PSP/FPS total score and of subscales are represented in Table 3; Figure 9 shows the frequency distributions of scores, in males and females.

The average total score is 55.0 ± 22.47 on a scale 0–100. The difficulties are mainly in the Work and Relationships area, with mean values corresponding to “evident” problems, and relevant or serious problems in about one half of the cases; the Personal and Health Care area shows a lower mean level of difficulties, and relevant or serious problems in about one third of the subjects.

It is important to highlight that the mean value of the Bother and Aggressiveness area is < 1, corresponding to a level of absent or mild difficulties; however, relevant or serious problems occurred in about 20% of males and 10% of females. Unfortunately, 3 patients were perpetrator of murder, 2 of them after interrupting the therapy.

In the common opinion there is a widespread false belief, amplified by the media, that all incidents of violence are due to insanity, and that all the mentally ill are violent: this is wrong [33] but any further violent accident, although not serious, reinforces and perpetuates this prejudice, whereas serious episodes of violence damage not only the persons injured or killed, but also all psychiatric users. Therefore, it is mandatory to prevent in every possible way any type of violence, stirred by the patients or, perhaps even more frequently, suffered by them.

The Personal and Social Functioning is significantly worse (p < 0.0001) in males than females, both in total score and in the areas of Social Relationships and Personal and Health Care. This corresponds to literature data: almost all the studies reviewed in 1997 [30] and the Maine and Vermont three decades studies [32] underlined better clinical outcome and better social adjustment in females.

In this research, the gender difference in the course of schizophrenia is significant: in addition to the worse functioning, males have an earlier age at T0 (about 7 years earlier, on average, across the entire sample, 5.5 years earlier in the cases under treatment); they have a significantly higher THI and AHI indexes, and a significantly greater number of CMHC interventions per person per year (p = 0.003). The difference seems to stem from specific sex differences in genetics, endocrinology or perhaps in structural brain characteristics [30], because it is not adequately explained by environmental factors, as currently in our society the availability and accessibility of psychiatric services are similar for both genders, as are their lifestyles. Moreover, the education level is higher in females than in males, and we do not know protective socio-cultural attitudes toward females; on the contrary, in the Italian society, they often undergo discrimination, namely in the working environment and they are generally required to show greater efficiency at home, at work and in social relationships.

These data indicate the need for programming, in males, both increased resources for the prevention of hospitalization and specific programs for the prevention of disability.

The analysis with the Spearman test of the correlations between the different parameters of course and outcome shows strong correlations (r ≥ 80) among all the admissions parameters: number and length of total admissions, number and length of admissions for acute symptoms, THI and AHI Indexes and length of residential rehabilitation programs, excluding only the number of admission for residential rehabilitation programs. These correlations may indicate that the subjects with high hospitalization are high users both in number and duration of hospitalizations, mainly for acute crisis, and that they also require residential rehabilitation programs of very long duration.

Between THI and AHI Indexes there is a medium correlation (0.40 < r < 0.80), indicating the existence of needs of hospitalization different from the acute symptoms.

There are strong correlations, as expected, among BPRS total score and BPRS subscales score, excluded the Anxiety-Depression subscale: they are related to the structure of the test, with the Anxiety-Depression scale less specific with respect to schizophrenia.

The total PSP/FPS score is strongly correlated with the subscale of Work and Socially Useful Activities, with a negative correlation, because the total score and the score of subscales, in this test, are inversely calculated. Medium negative correlations were found among the FPS total score and the other three subscales and among each of the subscales, except for the Bother/Aggressiveness subscale, which correlates only with the area of Self-Care/Health Care.

This can lead to some hypotheses: that work is the main problem in the functioning and/or that the work is the most important factor of evaluation in this test; that the level of personal functioning may differ significantly in different areas; that bother and aggressiveness may be partly related to poor health care and poor compliance.

It is interesting to note that the parameters of hospitalization show only weak correlations (r < 0.40) with the parameters related to symptoms (BPRS) and functioning (PSP/FPS) at present time.

It is even more interesting to note that non-significant correlations resulted between symptoms and functioning, and among the age at T0 and all the parameters of course and outcome; the same applies to the years of observation.

The data show that symptoms and functioning are not interdependent upon each other, nor strictly correlated to the time spent in hospital in the disease course; furthermore, clinical course and functioning, in our sample, are not affected by the early onset or by a long disease duration: even cases with early onset, high index of hospitalization, long time of disease can reach the remission of symptoms or a good level of functioning.

**Figure 10:**
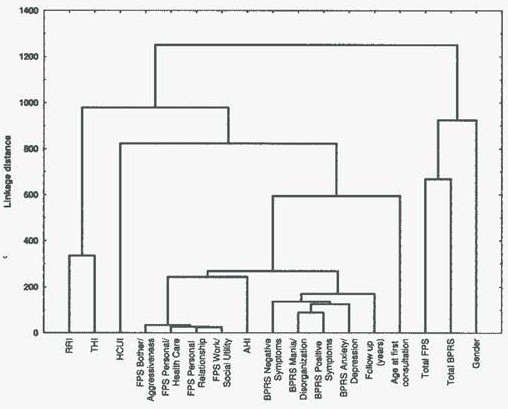
Cluster Analysis.

The results of the Cluster Analysis are shown in Figure 10: all the FPS subscales are grouped together, and the AHI index is unexpectedly joined to this group. The indexes of symptoms evaluation, i.e. the BPRS subscales, are grouped together and form a cluster with the time of observation. The THI and the RRI indexes form a cluster well separated from both the symptoms and functioning, and especially from the AHI index. A particular cluster is composed of PSP/FPS and BPRS total, which are particularly close to gender as highlighted by previous analyses, where the level of functioning and the symptoms resulted significantly different in the two genders.

The parameters of symptoms evaluation groups together and forms a cluster with the length of follow-up : this may indicate that the most symptomatic subjects are kept in contact with the Department and follow treatment programs of longer duration, in the context of good clinical practice. The particular relationship between negative symptoms and years of observation might indicate this area as the main problem of chronicity.

The close relationship between THI and RRI, but not with AHI, confirms the greater weight of the residential rehabilitative admissions over THI; but the THI and RRI Indexes are also well separated from symptoms and functioning, and this may suggest the hypothesis that the admissions in the rehabilitative-residential units seems to include a strong “residential” component. This may be linked to factors as housing needs or intra-familial disputes or environmental problems, rather than clinical needs or rehabilitation purposes. If this hypothesis will prove to be well founded, we will need to find alternative solutions to the “residential” needs, more appropriate and certainly less expensive than specialized rehabilitation units. These must be reserved to the specific interventions for which they were created.

In the Cluster Analysis, all the FPS subscales are grouped with each other and together with AHI: this may suggest that the main factor in the admissions into wards for acute symptoms is the disability rather than the symptomatology? Knowing that in this model of care the hospitalization is limited as much as possible for the shortest possible time, we can hypothesize that people with greater disability (low compliance included) are unable to fully utilize the opportunities of outpatient care, and this leads to higher AHI for exacerbations and relapses.

Strengths of the present research, in our opinion, are:

–  The sample, consisting of all the overt cases of a whole population living in a geographic area;

–  The length of the observation period, which is mandatory to assess trends in a disease so variable among individuals and for single individuals over time;

–  The observation of patients in real life, without artifacts of institutionalization;

–  The study of individual cases’ course, in addition to the study of the entire sample;

–  A stable model of care, minimizing “external” variability factors;

–  The study of present conditions for both symptoms and personal and social functioning.

Problematic aspects of this work are:

–  The difficulty of translating individual variability in comparable data;

–  The inability to reconstruct in all cases the different therapeutic paths including drug therapies and treatment programs: this forced us to postpone the study of these variables to a next phase, in more limited samples;

–  The incompleteness of some data, particularly: the causes of death, the day-centers data and several years of CMHC data, which were recorded and computerized, but unfortunately became inaccessible after moving from an information system to another.

A positive result of this work, not entirely expected, is the improvement of motivation of mental health professionals: reviewing the good results of past work can help them in the present task.

## Conclusions

The schizophrenia syndrome, in this research, appears as a disease treatable in outpatient care, with a low use of hospitalization, and without institutionalization: this allows considerable savings of public money, and has a positive impact on the life of users.

The clinical improvement is nowadays an achievable task and the remission is becoming a realistic goal, also in cases with early onset or a long history of disease.

The functioning is the main problem, at present time: we need to focus on rehabilitation and on socializing interventions and, even more, on the prevention of disability and of social exclusion.

The closure of psychiatric hospitals in Italy arose from a need to restore dignity to people rather than an evidence-based clinical choice; the results, after thirty years, have exceeded the expectations, mainly for schizophrenic cases, which accounted, and still do, for about half of the long-term assisted in the Department of Psychiatry. The Community Mental Health model, according to WHO recommendations [6] provides excellent clinical results, with lower costs and a greater respect for human rights. Much work remains to be done, aimed at improving not only the personal and social functioning, but also compliance, quality of life, active participation of users, social integration, and aimed to fighting the stigma still in existence. As a matter of fact, operators and user, are also well aware of the persisting prejudice and discrimination against mental illness and psychosis in particular, and realize that these preconceptions often prevent the timely recognition of symptoms, the demand for treatment at Mental Health Department and the correct execution of treatment program.

The data of this clinical research on the field clearly show the need to focus on home care instead of the hospital and strongly reaffirm the futility of the long-term psychiatric institutions, a dangerous temptation that unfortunately returns from time to time.

## Abbreviations

ICD: International Classification of Diseases; DSM: Diagnostic and Statistical Manual of Mental Disorders; BPRS: Brief Psychiatric Rating Scale; PSP: Personal and Social Performance Scale; FPS: Social and Personal Functioning; SOFAS: Social and Occupational Functioning Assessment Scale; THI: Total Hospitalization Index; AHI: Acute Hospitalization Index; RRI: Residential Rehabilitation Index; CMHC: Community Mental health Centre; SD: Standard deviation; IR: Inter-quartile Range.

## Competing interests

The authors declare that they have no competing interests and that none of the researchers has received funding for this work.

## Authors’ contributions

MCT, GC, AG and MGN designed the work and collected all the data; CS and EC analyzed the data; MCT and GC blindly scored the BPRS and PSP/FPS tests; all together discussed and wrote the final version of the work. All authors read and approved the final manuscript.
